# Changes of heart rate variability and hemodynamics within the frontal cortex in response to face emotional stimulation

**DOI:** 10.1192/j.eurpsy.2025.540

**Published:** 2025-08-26

**Authors:** B. Nam, S. A. Chung, H. Hwang, H. J. Kim, J. Hong, S. M. Kim, D. H. Han

**Affiliations:** 1 Dr. Nam’s Psychiatric Clinic, Chungju; 2 Chung Ang University Hospital, Seoul, Korea, Republic Of

## Abstract

**Introduction:**

There is an increasing demand for alternative evaluation tools capable of providing objective assessments or highlighting differences. Functional near-infrared spectroscopy (fNIRS) and heart rate variability (HRV) are frequently employed as biomarkers for assessing emotional status.

**Objectives:**

This study hypothesizes that emotional expressions, particularly unpleasant emotions and their variations in adolescents, are associated with changes in heart rate variability and frontal lobe activity.

**Methods:**

A total of 55 adolescents participated in this study. Following the completion of clinical scales, assessments of both HRV and fNIRS in a resting state were conducted for all participants for 200 seconds. After a 10-second rest, HRV and fNIRS assessments were performed during a positive emotional perception test for 192 seconds. Following a 30-second rest, the same procedures were repeated during a negative emotional perception test.

**Results:**

The correction rate of unpleasant emotional perception negatively correlated with HRV measures (unpleasant-HF, unpleasant-SDNN) and positively with pleasant-RMSSD. Additionally, it positively correlated with the ΔaccHBO2 within the left dorsolateral prefrontal cortex (DLPFC). Conversely, the correction rate of pleasant emotional perception negatively correlated with increases in ΔaccHBO2 within the left DLPFC. Both unpleasant-SDNN and unpleasant-HF negatively correlated with ΔaccHBO2 within the left DLPFC.

**Conclusions:**

The perception of negative emotions in adolescents is associated with individual levels of depression and anxiety. Furthermore, the perception of negative emotions significantly correlates with changes in HRV and activity within the left DLPFC. There is also evidence suggesting a link between changes in HRV and brain activity in response to the perception of negative emotions.

**Disclosure of Interest:**

None Declared

